# Childhood trauma and problematic internet use: A meta-analysis based on students in mainland China

**DOI:** 10.3389/fpsyg.2023.1115129

**Published:** 2023-04-05

**Authors:** Xiaotong Wang, Dexian Li, Shunyu Li

**Affiliations:** ^1^School of Education, Liaoning Normal University, Dalian, Liaoning, China; ^2^Center for Teacher Education Research in Xinjiang, Xinjiang Normal University, Urumchi, Xinjiang, China

**Keywords:** childhood trauma, problematic Internet use, mental health of students, meta-analysis, moderating variables

## Abstract

**Background:**

In recent years, the relationship between childhood trauma and problematic Internet use has been widely studied by scholars, but the research conclusions on the relationship between them are not consistent. Some studies report that childhood trauma and problematic Internet use are significantly correlated. However, others believe that there is a weak correlation between them. So the relationship between them needs to be studied further. The aim of this study is to explore the relationship between childhood trauma and problematic Internet use, and the effects of some moderating variables on both for students in Mainland China.

**Methods:**

This study followed the requirements of Preferred Reporting Items for Systematic Review and Meta-analyses (PRISMA) statement for literature screening. We searched the related studies on childhood trauma and problematic Internet use in Mainland China from January 2009 to November 2022 from CNKI, Wanfang Data, Chongqing VIP Information Co., Ltd. (VIP), Baidu scholar, ProQuest dissertations, SAGE Online Journals, Elsevier SDOL, Taylor & Francis, Springer, Web of Science, Google Scholar, EBSCO, Medline, Scopus Database, PubMed Central, Embase, The Cochrane Library, PsycINFO, CMA 3.0 was used to analyse the overall effect and test the moderating effect.

**Results:**

Among the papers included in the meta-analysis in this study, 31 papers reflected the relationship between childhood trauma and problematic Internet use, involving 52,503 subjects, and the sample size ranged from 388 to 16,130. This relationship between childhood trauma and problematic Internet use can be considered as a moderate correlation (*r* = 0.281, 95%CI[0.221, 0.338], *p* < 0.001). The results showed that the relationship between childhood trauma and problematic Internet use was affected by different problematic Internet use measures. Meanwhile, meta-regression demonstrated that the relationship between childhood trauma and problematic Internet use was moderated by survey’s year. Specifically, the correlation coefficient between childhood trauma and problematic Internet use also increases with increasing year. However, the relationship between childhood trauma and problematic Internet use was not affected by the region, grade, childhood trauma measures, publication source.

**Conclusion:**

Childhood trauma is closely related to problematic Internet use. In order to reduce problematic Internet use, corresponding prevention and intervention measures should be taken.

## Introduction

In recent years, the continuous development of network technology has greatly facilitated people’s life, such as providing a good way for the growth of students, meeting their needs of learning, communicating each other and seeking a sense of belonging (Fang and Sun, 2017; López et al., 2017; Shen, 2022). However, excessive Internet use is likely to cause social problems, such as problematic Internet use (PIU). PIU also variously termed Internet addiction, pathological Internet use, computer addiction, compulsive Internet use, Internet mania, pathological Internet use, and Internet abuse (Li and Chung, 2006; Cao et al., 2011; Tokunaga and Rains, 2016; Ramón-Arbués et al., 2021; Diotaiuti et al., 2022). PIU individuals use the Internet for a long time, and if they stop using the Internet, they will have withdrawal symptoms which will result in obvious social and psychological impairment (Brenner, 1997; Young, 1998; Griffiths, 2000; Davis, 2001; Wang and Cheng, 2019; Arrivillaga et al., 2020). Some studies have found that PIU students have become more and more significant with the development and change of the society (Li et al., 2020, 2022), moreover, PIU will lead to some negative influences (students’ low emotional regulation ability, weak executive control, distractions, depression, anxiety, suicide, etc.; Wu et al., 2015; Kim et al., 2018; Karaer and Akdemir, 2019; Chen et al., 2020; Wang S. et al., 2020; Ramón-Arbués et al., 2021; Agus et al., 2022). Therefore, how to control and reduce students’ PIU has become a hot issue for scholars to study.

Previous studies explored the contributing factors and the formation mechanism of PIU based on environmental factors (home environment, the school environment) and personal factors (depression, social anxiety and stress; Ostovar et al., 2016; Jia et al., 2017; Stavropoulos et al., 2017; Li et al., 2018; Lei et al., 2019). According to the Interaction of person Affect Cognition (I-PACE) model, loneliness, depression, social anxiety, stress and other contents belong to person’s core characteristics, which is the inducement as well as important factors influencing the formation of PIU (Brand et al., 2016). In addition, childhood trauma (CT) is also a very essential factor of person’s core characteristics. According to the Schema Theory, early childhood experience is an important part of the formation of schemas, which helps people to complete cognitive function, while maladaptive schemas can be triggered by adverse early experiences such as CT, which will lead to Maladaptive behaviors such as addiction (Young et al., 2007). At present, I-PACE model and Schema Theory were confirmed by a large number of studies, indicating that CT was significantly correlated with PIU, and CT was an important predictor of PIU (Sharifi, 2019; Dong et al., 2021). Maybe their relationship is mediated by the subjects’ tendency toward temporal dissociation and cognitive absorption. Due to the fact that individuals who have suffered CT are more likely to perceive stress, which makes it easier to have temporal dissociation easily (perceive time as passing more slowly), and the inability to more accurately perceive the passage of time during a deeply engaged online experience increases the risk of developing PIU (Drakulić et al., 2018; Cannito et al., 2022; Holman et al., 2022). However, some studies have drawn conclusions inconsistently that there was a weak correlation between CT and PIU (Zhang et al., 2012; Jhone et al., 2021). It may be related to Social Support and Computer-mediated Communication Theory. According to this theory, the existence of social interaction depends on common characteristics and interests of individuals in computer-mediated environment, and the resulting social ties are closer. It means that the use of the Internet can provide social support, which plays a role in relieving stress for individuals, and helps sub-healthy individuals recover (Wright et al., 2003). Since the Internet could provide a platform for people who are also suffering from CT, they provide social support for each other, which plays a positive role in CT people and alleviate PIU, without limitation of time and place. Whether these inconsistent conclusions are caused by differences in sample size, gender, region, grade, survey’s year, measurement instruments and the publication source need to be further explored. Therefore, this study aims at explore the relationship between CT and PIU by meta-analysis, and hypothesized that the relationship would be moderated by measurement instruments, the publication source, and demographic variables (gender, region, grade, survey’s year).

### Measures of childhood trauma and problematic Internet use

CT, also called childhood maltreatment, adverse childhood experience (Zhang et al., 2020a,b), including physical abuse, emotional abuse, sexual abuse, neglect and other forms of harm that occurs in children under the age of 18 (World Health Organization, 2022). The research on CT originated from people’s concern about sexual abuse. Russell defined sexual abuse as one or more unwanted sexual experiences between an individual under 18 years of age and a related or unrelated person (Russell, 1983). Based on the definition of Sexual Abuse, Russell formulated the Sexual Abuse Questionnaire by asking 14 interview questions. Later, the meaning of CT became richer than before, including psychological abuse, physical abuse, neglect and other new connotations in addition to sexual abuse, resulting in a variety of tools for measuring. Childhood Trauma Questionnaire (CTQ) has a good effect among 52 CT questionnaires (Saini et al., 2019). The CTQ, including 70 items in four dimensions: physical, emotional abuse, emotional neglect, sexual abuse and physical neglect, was developed by Bernstein and colleagues to assess self-reported experiences of abuse and neglect during childhood and adolescence (Bernstein and Fink, 1998). In order to detect the CT condition of normal population and clinical population in a short time, Bernstein et al. reduced and adapted the initial CTQ items, divided the dimension “physical and emotional abuse” into “physical abuse” and “emotional abuse,” changed the original four rotated factors into five (Bernstein et al., 2003). The Childhood Trauma Questionnaire-Short Form (CTQ-SF) with only 28 questions has been developed, which has been widely verified in different groups (Bernstein et al., 1997; Garrusi and Nakhaee, 2009; Wingenfeld et al., 2010). Meng and Liu (1994) made the earliest research on CT in mainland China. They published a research review related to child abuse in 1994, and since then the issue of CT has been widely concerned by Chinese researchers (Deng, 2000; Li et al., 2004; Yang et al., 2004; Pan and Li, 2005). Among them, the earliest Questionnaire of CT was The Screen Questionnaire of Child Abuse, developed by Yang et al. (2004), according to the WHO definition of child abuse, the questionnaire included eight items, including verbal abuse, economic control, isolation, neglect, punching, kicking, biting and slapping, using knife and\or stick, and sexual assault. In the study of the relationship between CT and PIU, the Chinese version revised according to CTQ-SF by Zhao et al. is most commonly used by scholars in mainland China (Zhao et al., 2005). In addition, the Child Psychological Abuse and Neglect Scale (CPANS) compiled by Deng, a Chinese mainland scholar, was often used to detect CT (Liu, 2016; Yang et al., 2017; Du, 2020; Zeng, 2020). This scale named CPANS, based on the different definitions and types of psychological abuse and neglect in literature, contains psychological abuse sub-scales (scold, intimidation, interference) and ignore sub-scales (emotional neglect, education, body/supervision ignore). It is a five-point scale, from 0 (none) to 4 (always), Cronbach’s coefficient is above 0.8 (Deng et al., 2007).

Since the American psychiatrist Goldberg (1995) first proposed the term Internet addiction disorder (IAD) in 1995, scholars have carried out a series of exploratory studies in this field, and PIU measurement tools have been constantly emerging (Rial et al., 2015). In mainland China, the most widely used measurement tool for PIU research are Internet Addiction Test (IAT; Young, 2001), the Revised Chinese Internet Addiction Scale (CIAS-R; Chen et al., 2003), and Adolescent Pathological Internet Use Scale (APIUS; Lei and Yang, 2007). Young believed that the diagnostic criteria for pathological gambling were most closely related to the pathological features of PIU. Therefore, the DSM-IV pathological gambling criteria were revised to form IAT to measure PIU, including 20-item covering the degree to which their Internet use affects their daily routine, social life, productivity, sleeping pattern, and feelings, items are rated on a 5-point Likert scale, and the higher the score, the higher the degree of addiction (Widyanto and McMurran, 2004). CIAS-R is a self-rating scale, which was compiled by Chen et al. (2003), scholars from Taiwan, China, on the theoretical basis of the core symptoms of Internet overuse and the problems related to Internet overuse. The full scale contains five dimensions: compulsive Internet use, withdrawal behavior and withdrawal response, PIU tolerance, time management problems, interpersonal and health problems, a total of 26 items. The total score reflects the degree of personal Internet addiction, and the higher the total score, the higher the PIU tendency (Mak et al., 2014), the internal consistency coefficient of each factor in this measure is between 0.70 and 0.82, and the internal consistency coefficient of the whole scale is 0.92. APIUS was formulated by Lei and Yang, scholars from Mainland China, according to the definition and dimensions of PIU and with reference to the existing items of related scales. The full scale includes six dimensions (salient; tolerance; compulsive Internet use/withdrawal symptoms; mood change; social comfort; negative consequences) with 38 items. It is a five-point scale, the higher the score, the more serious the PIU. Additionally, some studies also use self-designed scales to measure PIU, such as Different Types of Internet Addiction Scale for Undergraduates, and An Internet Relationship Dependency Inventory (Qian et al., 2006; Zhou and Yang, 2006). Different tools have different theoretical bases and dimensions, so the use of different measurement tools to explore the relationship between CT and PIU may be prone to bias in a certain extent. Therefore, this study took measurement tools as moderating variables affecting the two for further analysis.

### Demographic variables as moderators

#### Gender

Gender difference may play a role in moderating CT and PIU. Previous research has found a significant relationship between CT and PIU in women, but not in men (Dong et al., 2021). But other studies have found that traumatic experiences predict PIU in men but not in women (Schimmenti et al., 2017), and studies have found a correlation between PIU and CT in both men and women (Yates et al., 2012). Therefore, this study would investigate whether gender difference moderates the correlation between CT and PIU.

#### Survey’s year

In addition to gender, changes in year may also moderate the relationship between CT and PIU. Previous study has found that the correlation between CT and PIU decreases by growing years (Hsieh et al., 2021), However, other studies have shown that the correlation between CT and PIU increases with increasing years (Zhang et al., 2012; Wei, 2014; Liu, 2016; Li, 2020). Therefore, this study would explore the differences in CT and PIU among students in different years.

#### Region

Region may also be a variable moderating the relationship between CT and PIU. According to the level of economic development, mainland China can be divided into coastal and non-coastal areas (Li et al., 2020). Some studies have found that there is a low positive correlation between CT and PIU in coastal areas (Hu et al., 2021), but other studies have found that there is a moderate positive correlation between CT and PIU in coastal areas (Hou et al., 2021b). In researches of non-coastal areas, some studies have found that CT and PIU are positively correlated to a low degree (Li Y. et al., 2021), while some studies have found that CT and PIU are positively correlated to a moderate degree (Ye et al., 2020; Cao et al., 2021). However, some studies also found a moderately high positive correlation between CT and PIU (Dong et al., 2021). Therefore, this study would explore whether different regions are moderates the correlation between CT and PIU.

#### Grade

Grade may also be a moderating variable affecting the relationship between CT and PIU. Some studies found that correlation between CT and PIU of college students was positively correlated to a low degree (Wei, 2014), while other studies found that correlation between CT and PIU of college students was positively correlated to a moderate degree (Qin et al., 2022). In the survey of middle school students, some studies found that there was a low positive correlation between CT and PIU (Zhang S. J., 2020), while others found that there was a moderate positive correlation between CT and PIU (Du, 2020). Therefore, this study would investigate the moderating effect of different grades on CT and PIU.

#### Source of publication

In addition to the above moderating variables, the source of publication is also a moderating variable affecting the relationship between CT and PIU. Some studies have found that there is a low positive correlation between CT and PIU of subjects in journal papers (Zhang et al., 2012), but there is also a moderate positive correlation between CT and PIU of subjects in journal papers (Yang et al., 2017). In the master’s thesis and doctoral dissertation, some studies found that there was a low degree of positive correlation between CT and PIU (Wei, 2014), but other studies found that there was a moderate degree of positive correlation between CT and PIU (Liu, 2016). Therefore, this study would investigate the moderating effect in journal papers and dissertation on CT and PIU.

## Methods

### Literature search

This study followed the requirements of Preferred Reporting Items for Systematic Review and Meta-analyses (PRISMA) statement for literature screening (Moher D et al., 2009). We used databases to search the related studies on CT and PIU in Mainland China from January 2009 to November 2022, including CNKI, Wanfang Data, Chongqing VIP Information Co., Ltd. (VIP), Baidu scholar, ProQuest dissertations, SAGE Online Journals, Elsevier SDOL, Taylor & Francis, Springer, Web of Science, Google Scholar, EBSCO, Medline, Scopus Database, PubMed Central, Embase, The Cochrane Library, PsycINFO. The main search terms for CT are “childhood abuse” OR “childhood maltreatment” OR “childhood trauma” OR “childhood sexual abuse” OR “childhood physical abuse” OR “childhood psychological abuse” OR “childhood emotional abuse.” The search terms for PIU are “Internet overuse” OR “problematic Internet use” OR “Internet addiction” OR “Internet dependence” OR “excessive Internet use” OR “compulsive Internet use” OR “Internet addiction disorder” OR “pathological Internet use.”

### Study inclusion and exclusion criteria

Inclusion criteria: (1) Only cross-sectional studies were included, longitudinal studies were not included; (2) well-defined studies related to CT and PIU were included, complex trauma studies were not included; (3) only studies that use CT or PIU scales as research tools were included; (4) the empirical quantitative studies includes the Pearson product–moment correlation coefficient or the *t*-value and *f*-value that could be converted into *r* were reported; (5) accurate sample size was reported; (6) the subjects in the literature are Chinese mainland students, including primary school students, junior high school students, senior high school students, higher vocational students and university students, excluding students from Hong Kong, China; Macau, China; Taiwan, China. (7) self-assessment studies; (8) studies written in English or Chinese; (9) when the data is published repeatedly, only one professional academic journal is taken.

**Figure 1:**
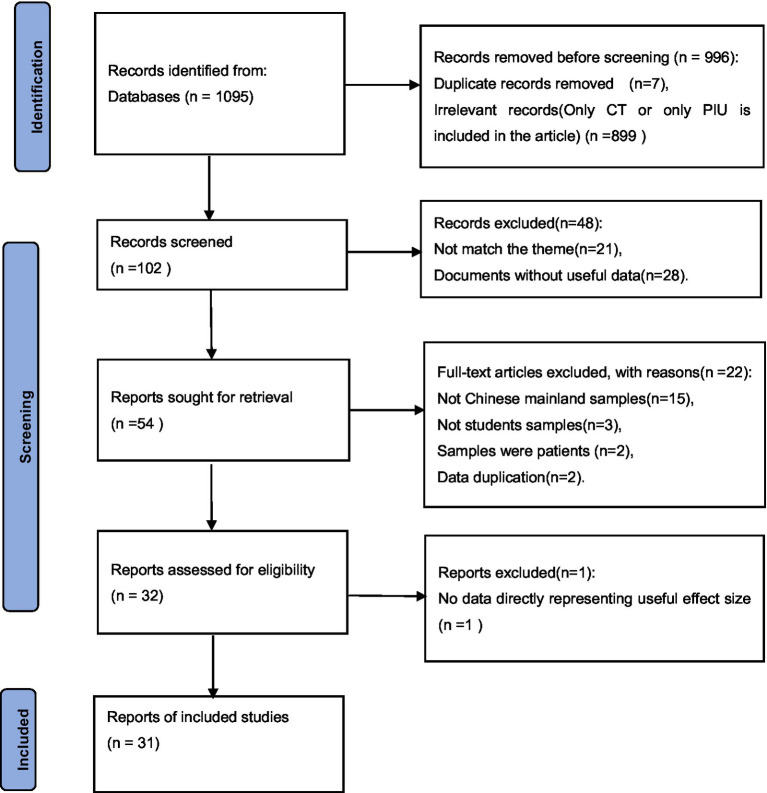
The PRISMA flow chart used to identify studies for detailed analysis of CT and PIU.

Exclusion criteria: (1) Incomplete reports of important data; (2) samples’ cognitive and judgment abilities were normal, and studies in which samples were patients. Excluding papers with no data, repeated publication and no clear sample size, a total of 31 papers met the meta-analysis criteria. Figure 1 depicts the PRISMA flow chart of the systematic search.

### Coding variables

The features of collected literature were coded, including the author’s name, publication time, survey’s year, source of publication, regional distribution, grade subjects, sample size and the percentage of the total number of girls, measuring tools, correlation coefficient of CT and PIU (see Table 1). Effect values were extracted according to the following criteria: (1) The correlation coefficient between CT and PIU was included in the coding; (2) Independent samples were coded once. If multiple independent samples were reported at the same time, they were coded separately; (3) When calculating the effect size of each category, each original datum appeared only once in each category to ensure the independence of the effect value calculation.

**Table 1:** Characteristics of the 31 studies included in the meta-analysis.

Number	Name (year)	Survey’s year	Source of publication	Region	Grade	*N*	Female %	PIU scale	CT scale	*r*
1	Cao et al. (2021)	2020	Journal article	Non-coastal	Younger	532	58.30%	APIUS	CTQ	0.360
2	Dong et al. (2010)	2008	Journal article	Coastal	Younger	1,193	44.68%	CIAS	CPANS	0.240
3	Dong et al. (2021)	2020	Journal article	Non-coastal	Undergraduate	1,749	49.90%	IAT	CTQ	0.764
4	Du (2020)	2018	Dissertation	Non-coastal	Younger	874	54.80%	IAT	CPANS	0.345
5	Hou et al. (2021b)	2020	Journal article	Coastal	Undergraduate	2,572	58.24%	APIUS	CTQ	0.410
6	Hou et al. (2021a)	2019	Journal article	Coastal	Undergraduate	1926	81.57%	APIUS	CTQ	0.268
7	Hu et al. (2020)	2018	Journal article	Non-coastal	Undergraduate	539	71.61%	CIAS	CTQ	0.230
8	Hu et al. (2021)	2020	Journal article	Coastal	Younger	439	48.89%	IAT	CTQ	0.149
9	Huang and Deng (2009)	2007	Journal article	Non-coastal	Undergraduate	740	81.89%	IAT	CPANS	0.110
10	Li (2020)	2018	Dissertation	Non-coastal	Undergraduate	949	59.50%	Others	CTQ	0.442
11	Li Y. et al. (2021)	2020	Journal article	Non-coastal	Undergraduate	1,178	67.70%	Others	CTQ	0.161
12	Liu (2016)	2014	Dissertation	Non-coastal	Younger	524	46.60%	APIUS	Others	0.381
13	Meng (2021)	2019	Dissertation	Non-coastal	Younger	1,117	51.83%	APIUS	CPANS	0.420
14	Peng et al. (2021)	2015	Journal article	Non-coastal	Younger	16,130	48.10%	IAT	Others	0.205
15	Qin et al. (2022)	2020	Journal article	Coastal	Undergraduate	918	56.97%	CIAS	CTQ	0.350
16	Shi et al. (2020)	2018	Journal article	Non-coastal	Undergraduate	922	51.50%	Others	CTQ	0.190
17	Shen (2022)	2021	Journal article	Non-coastal	Younger	844	49.3%	IAT	CTQ	0.250
18	Song (2013)	2012	Dissertation	Non-coastal	Undergraduate	1,106	39.69%	Others	CTQ	0.142
19	Wei (2014)	2012	Dissertation	Non-coastal	Undergraduate	829	61.28%	IAT	CTQ	0.180
20	Wei et al. (2020)	2018	Journal article	Non-coastal	Undergraduate	1,162	55.77%	IAT	CTQ	0.160
21	Wu et al. (2022)	2020	Journal article	Non-coastal	Younger	1,280	53.90%	Others	CPANS	0.250
22	Xie et al. (2021)	2019	Journal article	Non-coastal	Younger	779	48.91%	Others	CPANS	0.237
23	Yang et al. (2014)	2010	Journal article	Non-coastal	Younger	3,798	48.10%	IAT	Others	0.211
24	Yang et al. (2017)	2015	Journal article	Non-coastal	Undergraduate	388	70.10%	IAT	CPANS	0.416
25	Yang et al. (2021)	2019	Journal article	Non-coastal	Undergraduate	539	71.80%	CIAS	CTQ	0.220
26	Ye et al. (2020)	2018	Journal article	Non-coastal	Undergraduate	1,347	58.30%	Others	CPANS	0.360
27	Zeng (2020)	2018	Dissertation	Non-coastal	Younger	476	42.00%	Others	CPANS	0.200
28	Zhang M. (2020)	2018	Dissertation	Non-coastal	Younger	903	52.20%	Others	CTQ	0.230
29	Zhang S. J. (2020)	2018	Dissertation	Non-coastal	Younger	1,766	52.21%	Others	CTQ	0.171
30	Zhang (2022)	2020	Dissertation	Coastal	Younger	1,186	52.90%	Others	CTQ	0.315
31	Zhang et al. (2012)	2010	Journal article	Non-coastal	Younger	3,798	49.10%	IAT	Others	0.057

### Effect size calculation

The meta-analysis method of Person product difference correlation coefficient *r* was used to calculate the effect size in this study value (Borenstein et al., 2009). Fisher’s *Z* transformation was applied to *r*, and weights and 95% confidence intervals were calculated based on the sample size. Conversion formula: *Zr* = 0.5*ln[(1 + *r*)/(1 − *r*)], *VZ* = 1/*n* − 3, *SEz* = sqrt(1/*n* − 3), where *Zr* represents the converted value of the corresponding *r*, *VZ* is the variance, and *SEz* is the standard error.

### Data processing and analysis

For testing whether study result represents a estimated sample of the total effect size, a homogeneity test is required. Firstly, the homogeneity test provides a basis for whether the outcome adopts a fixed effect model or a random effect model. If the test results showed that the effect values were homogeneous, the fixed effect model was selected. If the heterogeneity is considerable, the random effect model should be used. Secondly, the homogeneity test also provides the basis for the analysis of the moderating effect, and the large heterogeneity indicates the existence of the moderating effect (Lipsey and Wilson, 2001).

## Results

### Effect size and the homogeneity test

Among the papers included in the meta-analysis in this study, 31 papers reflected the relationship between CT and PIU, involving 52,503 subjects, and the sample size ranged from 388 to 16,130. Table 2 shows the homogeneity test of CT and PIU in 31 independent samples, with *Q* statistic value of 1,499.887, *p* < 0.001*, I*^2^ = 98.000, indicating the heterogeneity of the included literature. This may be due to the use of different measurement tools, sources of subjects and different sample sizes in the literature, that is, there may be a moderating effect. According to the method provided by Lipsey and Wilson (2001), the included papers are highly heterogeneous and must be analyzed by random models.

**Table 2:** Random model of the correlations between CT and PIU.

*k*	*N*	Mean *r* Effect size	95% CI for *r*	Homogeneity test			Tau-squared			Test of null (two tailed)
				*Q*(*r*)	Value of *p*	I-squared	Tau-squared	SE	Tau	*Z*-Value
31	52,503	0.281	[0.221, 0.338]	1499.887	0.00	98.000	0.032	0.014	0.178	8.846***

*p < 0.05, **p < 0.01, ***p < 0.001, the same as follows.

A random model was used to analyze the correlation between CT and PIU, and it was found that CT was significantly correlated with PIU, with a correlation coefficient of 0.281, 95%CI[0.221, 0.338]. This relationship between CT and PIU can be considered as a moderate correlation (Lipsey and Wilson, 2001). The *Z*-value of the relationship between CT and PIU was 8.846, *p* < 0.001, indicating that the relationship between CT and PIU was stable.

### Moderator analysis

As mentioned above, random effects models should also be used in mediating effects analysis. Meta-ANOVA analysis is suitable for analyzing the moderating effects of categorical variables, such as the type of measurement tools, source of literature publication, regional distribution, and grade of subjects. In contrast, meta-regression analysis is suitable for analyzing the moderating effect of continuous variables, such as proportion of females and survey’s year.

### Meta-ANOVA analysis

In order to analyze the moderating effect of the relationship between CT and PIU, Meta-ANOVA analysis was used to analyze the moderating effect of categorical variables (Table 3). In terms of region, the results of homogeneity test (*Q* = 0.111, *df* = 1, *p* > 0.05) showed that region did not have a moderating effect on this correlation, and the relationship between CT and PIU was not affected by region.

**Table 3:** Meta-ANOVA analyses of region, grade, measures, and source of publication.

	Between-group effect (*Q_BET_*)	*k*	Mean *r*	effect size	SE	95% CI for *r*	Homogeneity test within each group (*Q_W_*)
					LL	UL	
**Region**	0.111						
Coastal		6	0.295	0.006	0.223	0.363	58.432***
Non-coastal		25	0.278	0.018	0.206	0.347	1394.484***
**Grade**	0.769						
Undergraduate		15	0.309	0.028	0.186	0.422	1032.159***
Younger		16	0.252	0.005	0.208	0.295	230.994***
**Measure’s of CT**	2.482						
CTQ		18	0.290	0.022	0.186	0.389	1075.404***
CPANS		9	0.289	0.006	0.223	0.353	82.927***
Others		4	0.209	0.008	0.122	0.292	97.726***
**Measure’s of PIU**	8.044*						
IAT		11	0.279	0.040	0.142	0.405	1224.200***
CIAS-R		4	0.263	0.004	0.199	0.325	10.876*
APIUS		5	0.368	0.006	0.302	0.432	34.513***
Others		11	0.248	0.005	0.190	0.304	112.812***
**Source of publication**	0.023						
Journal article		21	0.278	0.020	0.198	0.354	1356.857***
Dissertations		10	0.286	0.008	0.213	0.356	136.203***

In terms of grade, the results of homogeneity test (*Q* = 0.769, *df* = 1, *p* > 0.05) showed that grade did not have a moderating effect on this correlation, and the relationship between CT and PIU was not affected by grade.

As for the measurement tools of CT, the results of homogeneity test (*Q* = 2.482, *df* = 2, *p* > 0.05) showed that there was no moderating effect of the measurement tools of CT on this correlation, and the relationship between CT and PIU was not affected by the measurement tools of CT.

As for the measurement tools of PIU, the results of homogeneity test (*Q* = 8.044, *df* = 3, *p* < 0.05) showed that the measurement tools of PIU had moderating effect on the correlation, and the relationship between CT and PIU was affected by the measurement tools of PIU.

In terms of publication source, the results of homogeneity test (*Q* = 0.023, *df* = 1, *p* > 0.05) showed that there was no moderating effect of publication source on this correlation, and the relationship between CT and PIU was not affected by publication source.

### Meta-regression analysis

To examine whether continuous variables (gender and survey’s year) moderate the effect sizes between CT and PIU, the *r* effect size was meta-regressed onto the percentage of female participants and survey’s year in each sample. In Table 4, meta-regression (Q*_Model [1, k = 31]_* = 0.03, *p* > 0.05) demonstrated that there was no moderating effect of gender in the relationship between CT and PIU. Meta-regression (Q*_Model [1, k = 31]_* = 4.43, *p* < 0.05) demonstrated that the relation between CT and PIU was moderated by survey’s year. It means with the increase of year, the correlation coefficient between CT and PIU also increases.

**Table 4:** Meta-regression analysis of gender and survey’s year.

Variables	Parameter	Estimate	SE	*Z*-value	95%CI for b	
					LL	UL
Female (%)	*β_0_*	−0.0600	0.3239	−0.19	−0.6948	0.5748
	*β_1_*	0.3218	0.1845	1.74	−0.0398	0.6834
	*Q*_Model_ (1, *k* = 31) = 0.03, *p* > 0.05					
Survey’s year	*β_0_*	0.0159	0.0075	2.10	0.0011	0.0306
	*β_1_*	−31.7019	15.2041	−2.09	−61.5013	−1.9025
	*Q*_Model_ (1, *k* = 31) = 4.43, *p* < 0.05					

### Publication bias

**Figure 2:**
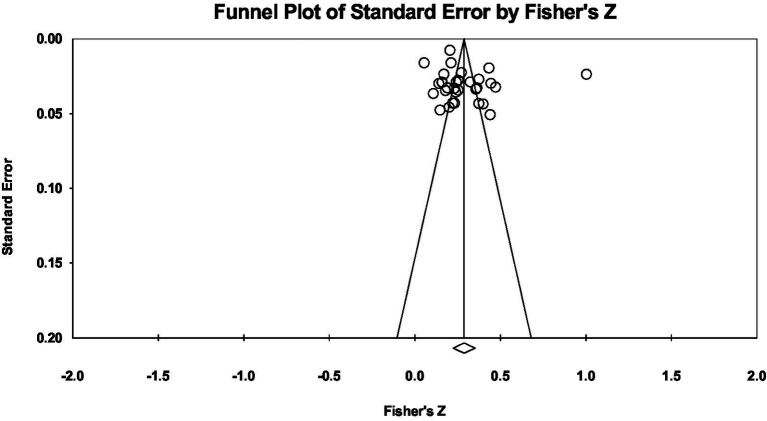
Funnel plot of effect sizes of the correlation between CT and PIU.

To examine whether the results were biased due to effect sizes from various sources, a funnel plot was drawn, indicating that the 31 effect sizes were symmetrically distributed on both sides of the average effect size, and an Egger’s regression (Egger et al., 1997) revealed no significant bias (*t_(29)_* = 1.173, *p* = 0.250 > 0.05). This result showed that in the overall correlation between CT and PIU was stable in this study (Figure 2).

## Discussion

### Relationship between childhood trauma and problematic Internet use

Based on 52,503 students in mainland China from 31 articles, the results of a meta-analysis showed that a moderate Holmanpositive correlation between CT and PIU, consistent with previous research results (Hou et al., 2021a; Qin et al., 2022; Zhang, 2022), it means that students who have experienced CT are more likely to suffer from PIU (Sharifi, 2019; Li, 2020). It supports the Interaction of Person-Affect-Cognition-Execution (I-PACE) model (Brand et al., 2016), according to this model, person’s core characteristics, including biological genetic and further bio-psychological factors, are the predisposing factors for the formation of PIU. In addition, moderating variables (including the individual’s subjective perception of the situation, poor coping style, as well as cognitive biases on the Internet, etc.), will lead to the desire of individuals to use the Internet, and then weaken the inhibitory control and the executive function, resulting in the decision to use the Internet. When individuals gain satisfaction in this process, it will strengthen the Internet use behavior, and then lead to PIU (Brand et al., 2016). It can be said that CT belongs to further bio-psychological factors, which is a predisposing factor to the formation of PIU. Since CT groups are more likely to perceive stress (McLaughlin et al., 2009; Edalati et al., 2019), this makes CT students unable to perceive the passage of time in the process of using the Internet, resulting in temporal dissociation and cognitive absorption (Drakulić et al., 2018; Cannito et al., 2022; Holman et al., 2022), thus, the cognitive process changes resulting in dysfunction, impulsivity and other adverse coping styles (Young et al., 2007; Perlman et al., 2016; Wang S. et al., 2020). Additionally, various factors such as “Internet related cognitive bias” (i.e., happiness or escapism through using the Internet) generated in the process of using the Internet become the moderating variables for forming PIU. When the CT people have problems such as anxiety, depression, aggression, social skills deficit and poor interpersonal relationships (Romano et al., 2014), the characteristics of Anonymity, Convenience and Escape of the Internet (Young, 1997) will produce positive associations and trigger their desire to use the Internet. The emergence of desire will weaken their own executive power and control, and make the decision about using the Internet. When the use of the Internet is satisfied and compensated, this process will be strengthened, increasing the frequency of Internet use, and then PIU will emerge. Therefore, individuals with traumatic childhood experiences are more likely to suffer from PIU.

### Moderating effects

#### The moderating role of PIU measures

Studies have shown that the relationship between CT and PIU is affected by different PIU measures. When the APIUS was used, the correlation coefficient between CT and PIU was bigger than that when other scales were used. This may be caused by the following reasons: APIUS was compiled by scholars in mainland China based on IAT and CIAS, combined with the actual situation of adolescents in mainland China, and integrated the cognitive, emotional and behavioral symptoms (Lei and Yang, 2007). Therefore, compared with other PIU measuring tools, APIUS can better adapt to the characteristics of students in mainland China. This supports the view of previous scholars that the measurement tools developed by local scholars are more culturally adaptable to native subjects (Lei et al., 2018). This suggests that the relationship between CT and PIU may be moderated by the suitability of PIU questionnaires, resulting in a higher correlation by using APIUS in studies.

#### The moderating role of survey’s year

The study showed that survey’s year was also a variable that mediated the relationship. The relationship between CT and PIU increased gradually with increasing years. This may be due to the following reasons. On the one hand, the number of people who experienced CT and suffered from adverse outcomes increased with increasing years (Statista Research Department, 2023). Studies have proposed that family is a high risk factor for child abuse (Miyamoto et al., 2017). With the development of society and the gradual formation of multicultural forms, dysfunctional families such as divorce, separation and single-parent families have increased, which directly or indirectly triggered CT (Xie, 2019). On the other hand, with the increase of the year, Internet technology has been constantly developed and improved, the emergence of phone watches, smart phones, smart TVs and other devices makes it more convenient for students to use the Internet. Additionally, many schools choose to take online courses at home during COVID-19, which also provides convenience for students to access the Internet (China Internet Network Information Center, 2022).These reasons may enhance the relationship between CT and PIU.

#### Implications of the study

The results of meta-analysis between CT and PIU suggest that effective prevention and intervention measures should be taken to reduce PIU. On the one hand, preventive measures need to be strengthened. First of all, the relationship between CT and PIU is moderate positive correlation. In order to reduce PIU, CT prevention should be strengthened. A good CT screening mechanism could identify CT groups in need of help timely and lay the foundation for subsequent intervention and treatment for them, thereby reducing the risk of CT (Gilgoff et al., 2020). However, there is no common CT screening mechanism (Sekhar et al., 2018). Therefore, it is necessary to develop CT screening mechanism based on evidence and theory, moreover, CT has intergenerational transitivity (Narayan et al., 2021), so it is necessary to improve the CT screening mechanism of parents further. In addition, families and schools have been turned out to be vital places for CT prevention (Yen et al., 2007; Chafouleas et al., 2018). School workers should take active approaches to create a safe and supportive atmosphere, as well as pay attention to CT individuals proactively (Chafouleas et al., 2018), and in families, parental education which is beneficial to strengthen the ties through useful communication between parents and their children, needs to be improved. A healthy interaction is good for helping kids reduce bad behaviors (Throuvala et al., 2019). Furthermore, strengthen the cooperation between families and schools to prevent CT in students (Mersky et al., 2011), prevent CT as early as possible, so as to reduce the possibility of PIU. Second, PIU needs to be prevented directly. Adolescents should build self-esteem, remain optimism, and also enhance skills and abilities to prevent IA, such as maintaining self-control, promoting the emotion regulation and strengthening social interaction (Koo and Kwon, 2014; Shek et al., 2016). Meanwhile, the role of the school system, which takes the form to prevent PIU through the training of teachers and parents, educating students, and raising awareness to create a good environment and reduce the possibility of negative events, needs to be watched (Romano, 2014). In addition, the relevant policies are tightening up in various countries and regions so as to prevent PIU among adolescents. The government of South Korea has launched Internet addiction prevention and treatment programs that cover people of all ages, including preschool children (Ministry of Science, ICT and Future Planning, and National Information Society Agency, 2013). The Ministry of Education of the People’s Republic of China, in cooperation with relevant departments, carries out special actions to control minors’ online environment, and adopts online game accounts and real-name systems on video platforms to prevent minors from indulging in the Internet (Ministry of Education of the PRC, 2020).

On the other hand, PIU could be reduced in scaling up interventions. First, training on mindfulness, boosting self-esteem and building resilience should be good for individuals who have already suffered from CT. Studies have shown that good mindfulness, high self-esteem and good resilience can help CT students get rid of anxiety, depression, sleep disorders and other problems, so as to improve the self-compassion and mental health of CT children which alleviate the negative impact of CT (Joss et al., 2019; Chen and Qin, 2020; Fitzgerald, 2022). At the same time, schools should make some upgrades and service expansion for CT groups, like professionals provide support for children with CT, including psychological counseling and behavior modification training as well as improving students’ understanding of CT (Fitzgerald, 2022), so as to alleviate the pain caused by CT and reduce the occurrence of PIU. Secondly, intervention was carried out for individuals with PIU to strengthen self-control and self-esteem. Good self-control and self-esteem can help individuals alleviate the symptoms of PIU (Li S. et al., 2021; Li Y. et al., 2021; Liang et al., 2022). In addition, parents need to enhance their awareness of PIU so as to take measures to help children alleviate PIU symptoms (provide guidance and support for them, monitor their Internet use, regulate their Internet use behavior; Li et al., 2013; Malak et al., 2017). At the same time, studies have shown that the support from teachers can help to reduce the problem behaviors of adolescents such as PIU and smartphone dependence, and have a positive impact on their mental health (Jia et al., 2018). Therefore, teachers can actively understand the psychology of PIU students and provide them with help and guidance.

#### Limitations and future studies

In this study, Egger’s publication bias test was applied to the meta-analysis results. It was revealed that the included studies did not have any obvious publication bias and that the meta-analysis results were stable. This indicates that, compared with the results of studies based on a single sample group, our results were more reliable, more representative, and more authentic. However, there are also some limitations in our study objectively. Future work can focus on the following aspects. (1) Students from primary schools, middle schools and universities in mainland China were selected as samples in this study. Future research can explore the relationship between CT and PIU in different groups, such as workers and farmers. (2) The data of this study relies on the subjective self-evaluation of the subjects, and the future work can use more objective data from other-rating methods. (3) Gender, survey’s year, region, gender, the measurement tool and the source of publication were selected as moderating variables in this study, and other variables such as family economic status and parents’ education level could be selected in future research. (4) This study selected the data of subjects in mainland China, and future research can go further to explore the relationship between CT and PIU of subjects worldwide.

## Conclusion

Through Meta-analysis of 52,503 Chinese mainland students in 31 articles, we found that CT and PIU were positively correlated to a moderate degree. At the same time, the relationship between the two was moderated by PIU measures and survey’s year. The results showed that the relationship between CT and PIU was affected by different problematic Internet use measures. Meta-regression demonstrated that the relationship between CT and PIU was moderated by year. It means the correlation coefficient between CT and PIU also increases with increasing year. From the results of this review, we recommend that professionals, such as clinicians, consider PIU as a potential disorder associated with CT and pay more attention to individuals with PIU and CT. For patients with PIU, CT status should be assessed. For CT individuals, preventive measures should be taken to prevent PIU, and intervention should be performed when it does not develop. Therefore, this study indicated that preventive measures and intervention measures should be taken to reduce students’ PIU through the cooperation of individual students, family and school. Although our findings did not focus on intervention, we provided an interesting conclusion for future researchers that CT is related to PIU, and that reducing CT may also help reduce PIU in individuals. It could be said that the efforts of this study would be helpful to promote research on the mechanism, prevention and intervention measures of PIU.

## Data availability statement

The original contributions presented in the study are included in the article/supplementary material, further inquiries can be directed to the corresponding author/s.

## Author contributions

XW did the data collection, was the major contributor in writing the manuscript. SL conceived and designed the study, and analyzed the data. DL is a supervisor in whole study. SL and DL reviewed and edited the whole paper. All authors contributed to the article and approved the submitted version.

## Funding

This research was sponsored by the Project of Social Science Foundation of Xinjiang Uygur Autonomous Region (22CMZ018) and the Project of Center for Teacher Education Research in Xinjiang (ZK202232B) and the Project of Doctoral Research Startup Fund at Xinjiang Normal University (XJNUBS201908).

## Conflict of interest

The authors declare that the research was conducted in the absence of any commercial or financial relationships that could be construed as a potential conflict of interest.

## Publisher’s note

All claims expressed in this article are solely those of the authors and do not necessarily represent those of their affiliated organizations, or those of the publisher, the editors and the reviewers. Any product that may be evaluated in this article, or claim that may be made by its manufacturer, is not guaranteed or endorsed by the publisher.

## Abbreviations

PIU: problematic Internet use
I-PACE: the Interaction of Person-Affect-Cognition model
CT: childhood trauma
CTQ: Childhood Trauma Questionnaire
CTQ-SF: Childhood Trauma Questionnaire-Short Form
WHO: World Health Organization
CPANS: the Child Psychological Abuse and Neglect Scale
IAD: Internet addiction disorder
IAT: Internet Addiction Test
CIAS-R: the Revised Chinese Internet Addiction Scale
APIUS: Adolescent Pathological Internet Use Scale
DSM-IV: Diagnostic Manual Of Mental Disorders
CNKI: Chinese National Knowledge Infrastructure
